# Commencing Technical Clinical Skills Training in the Early Stages of Medical Education: Exploring Student Views

**DOI:** 10.1007/s40670-018-00657-2

**Published:** 2018-11-30

**Authors:** Josephine Seale, Madeleine Knoetze, Anita Phung, David Prior, Colin Butchers

**Affiliations:** GKT School of Medical Education, Simulation and Interactive Learning (SaIL) Centre, King’s College London, Shepherds House, London, SE1 1UL UK

**Keywords:** Undergraduate medicine, Technical clinical skills, Student views, Student experience

## Abstract

**Introduction:**

Medical schools are increasingly introducing technical clinical skills training from year 1. However, little research has determined students’ views of such training. This study compared the perceptions of student groups which received different levels of technical skills training during the early years of their undergraduate medical degree.

**Methods:**

Medical students from King’s College London’s Stage curriculum (*n* = 184) receiving 48 h of technical skills teaching and Phase curriculum (*n* = 94), receiving 12 h, voluntarily participated. A mixed methods design using a questionnaire and focus groups explored students’ views. Stage and Phase student questionnaire responses were compared using Mann Whitney *U* tests. Focus group transcripts underwent thematic analysis.

**Results:**

The majority of Stage (*n* = 169) and Phase (*n* = 68) students identified year 1 as the best time to commence technical skills training. For the majority of the technical skills taught, Stage compared to Phase students reported feeling more prepared to perform them. Thematic analysis identified three main themes: Role of technical skills teaching in the early stages of medical education, impact on students’ learning and factors to consider when designing a medical undergraduate technical clinical skills programme.

**Conclusions:**

The wide student support and positive impact of technical skills training on students’ perceived preparedness for carrying out the techniques taught advocates its addition to the first year of the undergraduate medical curriculum. The identification by students of specific components considered to be fundamental in the effective teaching of technical skills provides guidance when designing future undergraduate clinical skills training.

**Electronic supplementary material:**

The online version of this article (10.1007/s40670-018-00657-2) contains supplementary material, which is available to authorized users.

## Introduction

Many undergraduate medical programmes introduce students to the clinical environment and the requisite skills during the early stages of training, moving away from the traditional model of distinct pre-clinical and clinical years. The term ‘clinical skills’ encapsulates a variety of abilities including history taking, physical examination, practical and technical skills. In the absence of clear definitions, a number of skills could be categorised as more than one of these terms [1]. For example, physical examination skills are also frequently referred to as practical skills. For the purposes of the present study, we use the term ‘technical skills’ to refer to clinical skills with a clear practical element, excluding examinations, ranging from urinalysis to subcutaneous injections.

Research in relation to clinical skills training in the pre-clinical years has predominantly focused on human factors and the practicalities of physical examination through the use of patient contact. These experiences are well received by students [2–4] and have consistently been shown to increase students’ self-reported competence and confidence in subsequent patient encounters [5–7]. Whilst these skills are integral to clinical practise, there is also a need to ensure students are competent in performing a variety of technical skills, for example, blood pressure measurement and oxygen administration. In the UK, the medical governing body, the General Medical Council (GMC), provides recommendations as to the technical skills students should be competent in at different stages of their degree [8]. Accordingly, many UK medical schools now commence technical clinical skills teaching from the first year of education in order to meet these recommendations. This competency-based approach to curriculum design is akin to other countries wherein similar skill lists are used to inform the development of medical curricula.

It may be postulated that initiating technical skills teaching in the first year of medical training provides students a longer time frame during which they can practise and develop these skills which, in turn, may be expected to result in increased confidence and preparedness to perform them in clinical practise. However, given the limited physiological and clinical knowledge of students at the start of their medical education, commencing training at this stage may have little benefit or relevance to the students. Research regarding students’ perceptions of early technical skills training and the potential impact on their perceived ability to perform the skills is currently lacking.

The undergraduate medical curriculum at King’s College London (KCL), UK, has undergone significant development culminating in the formation of the Stage Curriculum to replace the original Phase programme. One significant change has been the introduction of a technical clinical skills module in the first year of medical school. Based upon guidance from the GMC [8], students are taught techniques ranging from handwashing to oxygen administration**.** The Stage programme comprises of 48 h of this teaching during year 1 compared to the 12 h provided to students over the first 2 years of the Phase programme.

The present study aimed to explore student perceptions of technical skills training in the early stages of their medical education by comparing the views of two student groups with different levels of such training.

## Method

### Participants

A total of 184 year 1 students undertaking the Stage curriculum and 94 year 2 students undertaking the Phase curriculum voluntarily participated in the study. Students were recruited using e-mails to their student accounts inviting them to participate in the online survey and/or a focus group. A total of three recruitment e-mails were sent over a 2-week period.

### Study Design

The study was conducted on the KCL medical school campus during April 2017 prior to student summative examinations. Year 1 students on the Stage curriculum had received 48 h of predominantly face-to-face teaching on technical skills considered appropriate for their level of training by the GMC [8]. Sessions comprised of a lecture component related to the skill, a demonstration and the opportunity for students to practise and receive feedback. Five of the 48 h consisted of mandatory e-learning in which students worked through material related to the skill and completed a formative quiz to check their understanding. In addition, all students had six integrated sessions involving a simulated patient wherein they practised both patient communication and carrying out a previously taught technical skill. Students on the Phase programme received 12 h of similarly designed skills teaching over 2 years. An outline of the technical skills taught in each programme is provided in Table 1. All tutors were healthcare professionals who were knowledgeable and experienced in the skills being taught. Each participant was invited to complete an online questionnaire and attend a focus group. The study received ethical approval from the King’s College Research Ethics Committee (Number: LRS16/17-4300).

**Table 1:** Technical clinical skills taught during year 1 of the Stage curriculum and during years 1 and 2 of the Phase curriculum at KCL. BMI body mass index, NEWS National Early Warning Score, SBAR Situation, Background, Assessment, Recommendation

Stage curriculum Class-based sessions: 43 h e-learning: 5 h	Phase curriculum Class-based sessions: 12 h
Hand washing Instructing patients in the use of devices for inhaled medication† Interpretation of a NEWS chart Intramuscular injections Measuring and interpreting BMI*† Measuring blood glucose*† Measuring blood pressure† and pulse Measuring oxygen saturation Measuring respiratory rate Measuring temperature Oxygen administration Peak flow measurement† Prescribing (drug charts) Putting on sterile gloves SBAR handover Setting up a sterile field Subcutaneous injections Urinalysis†	Hand washing Instructing patients in the use of devices for inhaled medication Intramuscular injections Measuring blood pressure and pulse Measuring respiratory rate Measuring temperature Measuring oxygen saturation Peak flow measurement Putting on sterile gloves Setting up a sterile field Subcutaneous injections Urinalysis

*e-Learning session

†Included in an integrated scenario

### Data Collection

#### Questionnaire

Researchers developed the questionnaire in order to determine student views regarding the provision of technical clinical skills teaching during the early stages of the curriculum and their subsequent preparedness to carry out the skills they had been taught (Online Resource 1). An expert panel consisting of medical educators and clinicians was used to assess construct validity. Piloting the questionnaire with year 3 students demonstrated good internal reliability (Cronbach’s alpha = 0.9). A 5-point Likert scale of five items determined students’ views regarding the introduction of clinical skills training during the first year of medical education. A separate 5-point Likert scale of eight items ascertained students’ perceived preparedness to perform the technical skills previously taught in their sessions. The questionnaire was completed anonymously and accessed via a website link using the online survey software ‘SurveyMonkey’ (SurveyMonkey Inc., CA, USA).

#### Focus Groups

A total of eight focus groups comprising of a mix of Stage and Phase students further explored student views of the technical skills teaching they had received. All focus groups were facilitated by members of the research team. Following student consent, each focus group was audio-recorded and lasted approximately 1 h.

### Data Analysis

Questionnaire responses were analysed using SPSS version 22.0 (SPSS Inc., Chicago, USA). Stage and Phase student responses for each of the Likert scale items were compared using Mann-Whitney *U* tests. A significance level of *p* < 0.05 was applied. Audio recordings from each focus group were transcribed verbatim and underwent thematic analysis. A list of emerging themes was constructed, discussed and further developed by the research team. A consensus was reached on three main themes and all transcripts were coded accordingly.

## Results

### Questionnaire Results

A total of 184 year 1 Stage students and 94 year 2 Phase students completed the questionnaire resulting in a response rate of 41% and 25% respectively. Age ranged from 18 to 30 years with a male:female ratio of 1:2.

**Fig. 1:**
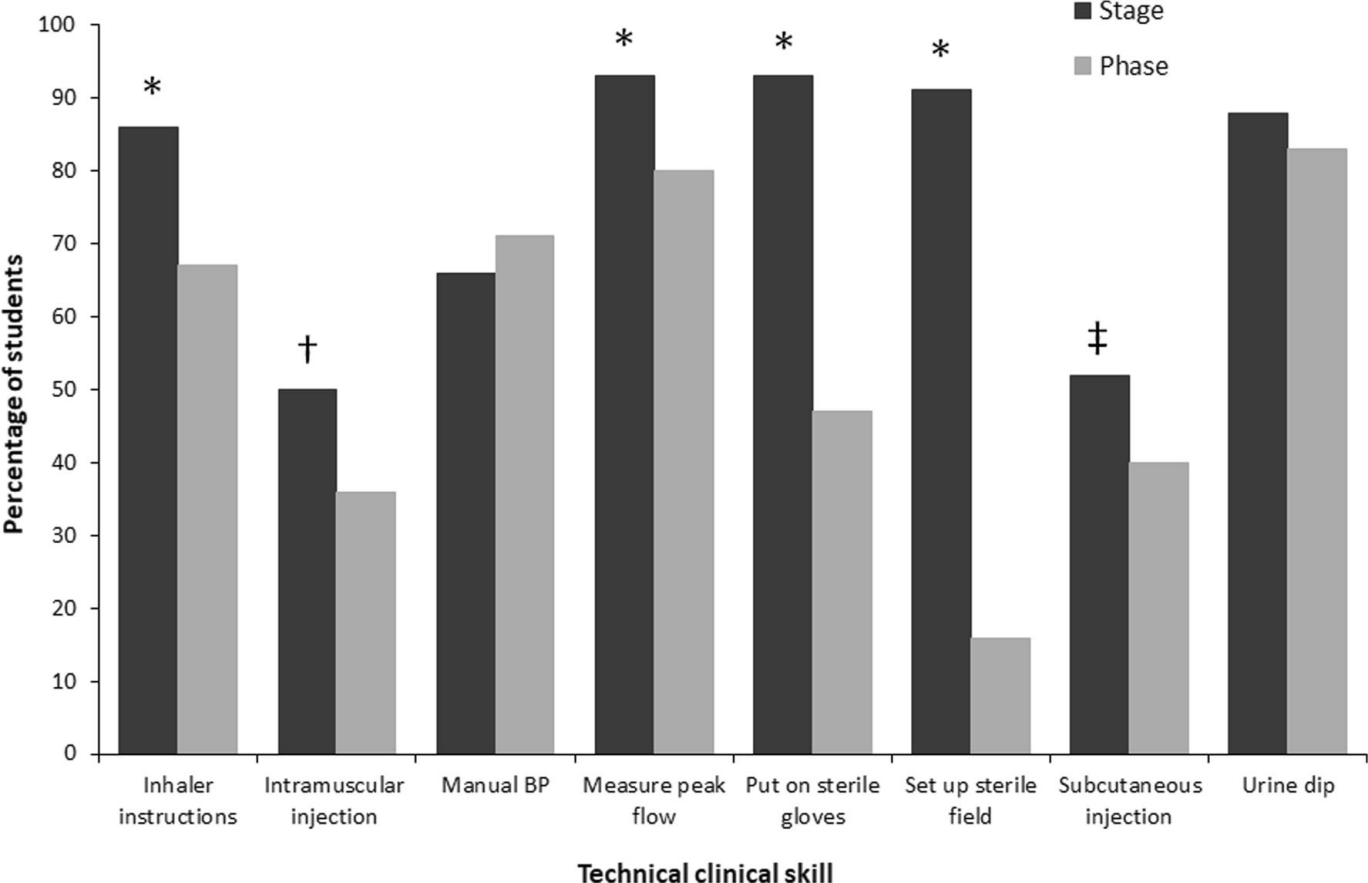
Percentage of Stage (*n* = 184) and Phase (*n* = 94) students who felt prepared or extremely prepared to carry out each technical clinical skill. BP, blood pressure. Asterisk indicates *p* < 0.001; dagger symbol indicates *p* < 0.01; double dagger symbol indicates *p* < 0.05

A high proportion of the Stage students (*n* = 169, 92%) and to a lesser extent Phase students (*n* = 64, 68%) who responded to the questionnaire thought year 1 was the best time to initiate technical skills training. The remaining respondents considered year 2 (Stage *n* = 13, 7%; Phase *n* = 29, 31%) or year 3 (Stage *n* = 2, 1%; Phase *n* = 1, 1%) as the most appropriate time to commence such training. Significantly, more Stage compared to Phase students reported feeling prepared to carry out all of the technical skills listed except for performing a urine dipstick test and taking a manual blood pressure measurement in which both groups reported similar levels of preparedness (Fig. 1).

Significantly, more of the Phase compared to Stage students completing the questionnaire reported that they did not receive enough technical skills teaching (*U* = 1820, z = − 11. 2, *p* < 0.001) or the opportunity to practise (*U* = 3170, z = − 8.89, *p* < 0.001). Both groups of students agreed that learning skills in a practical session enabled better understanding (Stage *n* = 182, 99%; Phase *n* = 94, 100%) and retention (Stage *n* = 178, 97%; Phase *n* = 90, 96%) of the topic than in a lecture.

### Focus Group Thematic Analysis

Eight focus groups were conducted with a total of 26 Stage and 20 Phase students. Thematic analysis identified three main themes: Role of technical skills teaching in the early stages of medical education, impact on students’ learning and factors to consider when designing a medical undergraduate technical clinical skills programme. Each theme comprised of a number of sub-themes.

#### Theme One: Role of Technical Skills Teaching in the Early Stages of Medical Education

##### Contextualisation

An advantage cited by students of commencing technical skills training during the first year of medical curriculum was that such learning broke up the predominantly didactic elements of the course and helped contextualise the knowledge gained from lectures, both of which were considered to aid their learning:

‘It’s really nice to be able to apply the things you learn in lectures’ [Phase student]

Nine Stage and six Phase students reported that early skills training made learning medicine more ‘real’, enabling them to appreciate that they were training to be a doctor as opposed to another profession:

‘Clinical skills teaching… puts it back into the context of you’re doing medicine not a natural sciences degree’ [Stage student]

‘Reminds you why you came to medical school’ [Phase student]

##### Relevancy

Twenty-two Stage and 12 Phase students reported that the skills taught were relevant. Four Stage and seven Phase students said that many technical skills were superfluous in the presence of increased automation with regard to healthcare measurements. In addition, three Phase students raised doubts regarding the extent to which students would have the opportunity to apply the skills they had been taught when on clinical placement:

‘The skill sets that the first years have mentioned….it’s unlikely you’re going to do that as a 2nd or 3rd year medical student’ [Phase student]

##### Preparation for Clinical Placement

In each focus group, more Stage compared to Phase students reported feeling prepared to perform the clinical skills they had been taught. The opportunity to practise was perceived by both student groups as a key factor in determining their sense of preparedness.

‘I feel more prepared than if we hadn’t had the lessons at all.’ [Stage student]

Stage and Phase students during each focus group expressed a mixture of opinions with regard to their perceived confidence to carry out the skills they had learned with some students feeling confident and others less so. Both groups overwhelmingly agreed that confidence was something an individual gained through experience, especially that related to real-life situations:

‘Are we ever really confident until we’ve done it in actual real life?’ [Stage student]

#### Theme Two: Impact on Students’ Learning

##### Motivation to Learn

Acquiring the ability to perform the technical skills carried out by medical staff was often cited by students from the Stage curriculum as the main motivation to learn the skills. Additionally, nine Stage students specifically reported that the technical skills sessions motivated further learning in relation to other areas of the course:

‘I found it motivating... it made me go and read about the subjects in the lectures and practise the skills’ [Stage student]

In contrast, 15 Phase students highlighted the upcoming Objective Structured Clinical Examinations (OSCEs) as their main motivation to learn the skills:

‘I feel forced to practise because I wouldn’t pass my exams if I didn’t practise’ [Phase student]

Only two Phase students explicitly reported that a desire to learn the skills regardless of examination purposes was a motivating factor.

##### Retention of Acquired Skills

A minority of Stage (*n* = 2) and Phase (*n* = 6) students raised concerns regarding the ability to retain the technical skills knowledge acquired during their first year over the long term:

‘If it’s not revisited, you forget it quickly’ [Phase student]

In contrast, early exposure to technical skills was considered by the remaining students from both groups to optimise the time available to practise and subsequently develop and retain the skills taught.

#### Theme Three: Factors to Consider When Designing a Medical Undergraduate Technical Clinical Skills Programme

##### Session Format

There was a widespread consensus by both groups with regard to the preferred format of technical skills sessions. Specifically, students requested background information about the skill, a practical demonstration by the tutor and then a significant proportion of time in which to practise. Phase students in particular sought clear guidance at the end of each session with regard to carrying out the skill in an examination situation.

‘It’s good we are informed on the theory and then after have the hands on experience…that’s the important part because we can’t relate to theory if we don’t actually do it’ [Stage student]

##### Opportunities for Practise and Consolidation

The majority of Stage (*n* = 21) and Phase (*n* = 14) students cited that opportunities to practise the technical skills, both in sessions and throughout the medical curriculum, were an important component of such skills training, primarily to prevent skills being forgotten.

##### Teaching Staff

Knowledgeable, engaging tutors and standardised teaching with clear instructions were considered by both groups as key factors when delivering clinical skills sessions. The opportunity to have constructive informal feedback on their performance during sessions was specifically highlighted by Stage (*n* = 13) and Phase (*n* = 7) students as important. Stage students predominantly reported feedback to be beneficial in preventing the development of ‘bad habits’ prior to conducting the skills in the clinical environment. Phase students identified feedback as relevant with regard to aiding their preparation for upcoming examinations.

## Discussion

The initiation of technical clinical skills teaching during the first year of medical school was overwhelmingly supported by Stage students in this study. In contrast, there was less of a consensus between Phase students with a considerable minority electing year 2 as an ideal time to start. The possibility that the educational benefit of early technical skills training could be limited if provided at a time when students have minimal physiological knowledge has been previously suggested [2, 3] and was expressed by a minority of Phase students in the present study. The finding that no Stage student expressed such a view suggests that they did not consider a lack of prior knowledge to be a disadvantage. In contrast, it may be argued that providing structured technical skills teaching as early as possible would allow students a greater time frame in which to practise and further develop their skillset which, in turn, could translate into an increased sense of preparedness when entering the clinical environment. The greater perception of preparedness in carrying out the majority of the skills taught by the Stage compared to Phase students supports this proposition. It is not clear why Phase students in the present study only felt equally prepared as their Stage counterparts in measuring blood pressure and performing urinalysis. Such a finding may reflect the effectiveness of the sessions which originally taught these particular skills or may indicate a greater degree of student self-directed learning and subsequent preparedness of these skills prior to their upcoming examinations. It was of interest that the increased preparedness felt by Stage students was not accompanied by an increased sense of confidence in conducting the skills when compared to Phase students. However, in agreeance with previous research [9, 10], both groups reported that confidence was something an individual gained through experience especially that related to real-life situations. This not only demonstrates the importance of placements in enhancing student confidence but also the potential benefits of using simulation-based learning modalities in skills teaching in order to emulate clinical scenarios in a safe environment.

The present research identified a number of benefits associated with early technical skills training, one of which was to provide a contextual basis to the knowledge being acquired during concurrent modules. Additionally, such skills sessions inherently encompass a practical element and consequently support a more active learning environment. The beneficial effect of ‘learning by doing’ has been previously described [11] and is supported by this study in which students stated an increased ability to understand and retain information when using practical as opposed to lecture-based sessions. Finally, the motivational effect of early technical skills training on the students’ desire to further their knowledge and practise the skills demonstrates a positive impact of these sessions on future learning.

Exploration of student views in the focus groups identified a number of factors considered by students to be integral when designing a clinical skills session. The format of sessions has been previously identified as an important aspect of clinical skill sessions focusing on physical examinations [12]. In the present study, a brief lecture-based component in conjunction with a strong focus on the practical element, especially the opportunity to practise and receive constructive feedback, was considered to be the most effective format. Commencing skills training at an early stage in medical education will allow for a longer opportunity to both practise the skills and address any misunderstandings which may arise during the initial stages of learning [13]. The importance of practise opportunities throughout the medical curriculum to maintain proficiency has been demonstrated for blood pressure measurement [14], a situation which is arguably applicable to any procedure taught. Subsequently, the use of a facilitated practise session following the training of any skill should not be underestimated. There currently exists a discrepancy in the literature as to whether tutors specialised in a skill results in greater student learning compared to those without specialist knowledge or experience [15–17]. However, in this study, knowledgeable tutors who provided feedback were considered to be important factors related to teaching. The identification of useful feedback as a desired feature of skills sessions supports previous studies wherein the provision of feedback has been associated with enhanced learning by students during communication and clinical skills sessions [12, 18, 19].

There are a number of limitations to this study. The voluntary participation and involvement of a single medical school reduces the generalisability of the results. In addition, the lower response rate from the Phase students may impact on the extent to which their responses are reflective of the entire cohort. The demonstration that self-reported ability is unreliable when compared to actual practise [20, 21] raises doubt as to whether the perceived preparedness in the current study will translate into enhanced clinical performance. Follow-up studies of the current student cohort will be required to clarify this issue. Finally, the present study was conducted prior to examinations in order to prevent any impact summative exam performance may have had on subsequent survey answers. However, Phase compared to Stage students were due to undertake a summative OSCE in the following month, a situation which may have influenced their perceptions of their clinical skills training. For example, only two Phase students highlighted that a desire to learn the skills regardless of examination purposes was a motivating factor. It may be that in the absence of impending OSCEs, more Phase students would have expressed this opinion.

## Conclusion

The results from this comparative study provide clear student support for the initiation of technical clinical skills training during the first year of medical school and demonstrate the impact such tuition can have on a students’ motivation to learn and preparedness to carry out the techniques taught. The request by both student groups for more practise and feedback opportunities in conjunction with knowledgeable tutors and a predominantly practical-based teaching session provides guidance when constructing future clinical skills sessions.

## Electronic supplementary material

ESM 1 (DOCX 21 kb)
