# Supplementary material for: Commencing Technical Clinical Skills Training in the Early Stages of Medical Education: Exploring Student Views
Source: Med Sci Educ. 2018 Nov 30;29(1):173–9. doi: 10.1007/s40670-018-00657-2 (PMC8368689; doi:10.1007/s40670-018-00657-2)
Supplement: Supplementary file 1 — (DOCX 21 kb) [file 40670_2018_657_MOESM1_ESM.docx]

Online Resource 1: Student questionnaire

| **General Information** | | | | | |
| --- | --- | --- | --- | --- | --- |
| What is the current year of your medical degree? □ Year 1 □ Year 2  Gender □ Male □ Female □ Prefer not to answer  Age range □ 18-20 □ 21-25 □ 26-30 □ 31+ □ Prefer not to answer  Are you a postgraduate? □ Yes □ No  Are you an international student? □ Yes □ No | | | | | |
| **At the present time how do you feel with regard to carrying out the following technical* clinical skills?**  ***Technical clinical skills do not include physical examination or history taking skills** | | | | | |
|  | **Extremely**  **Prepared** | **Prepared** | **Neither Prepared or Unprepared** | **Unprepared** | **Extremely Unprepared** |
| Performing a urine dip |  |  |  |  |  |
| Instructing a patient how to use an inhaler |  |  |  |  |  |
| Taking a manual blood pressure |  |  |  |  |  |
| Handing over a patient using SBAR (Situation, Background, Assessment, Recommendations) |  |  |  |  |  |
| Taking a peak flow measurement |  |  |  |  |  |
| Giving a subcutaneous injection |  |  |  |  |  |
| Putting on sterile gloves |  |  |  |  |  |
| Calculating a patient’s NEWS (National Early Warning Score) |  |  |  |  |  |
| Asking a patient for a urine sample |  |  |  |  |  |
| Setting up a sterile field |  |  |  |  |  |
| Writing a prescription on a hospital drug chart |  |  |  |  |  |
| Administering an intramuscular injection |  |  |  |  |  |
| Administering oxygen to an acutely unwell patient |  |  |  |  |  |
| **Please indicate the extent to which you agree/disagree with the following statements** | | | | | |
|  | **Strongly Agree** | **Agree** | **Neither**  **Agree or Disagree** | **Disagree** | **Strongly Disagree** |
| During year one I did not have enough teaching on technical skills |  |  |  |  |  |
| During year one I did not have enough opportunity to practise technical clinical skills |  |  |  |  |  |
| Learning about technical clinical skills is useful during year one |  |  |  |  |  |
| Learning technical skills in a practical session compared to a lecture enables better understanding of the topic |  |  |  |  |  |
| Learning technical skills in a practical session compared to a lecture enables better retention of the topic |  |  |  |  |  |
| **Your Views on Technical Clinical Skills Teaching During Year One** | | | | | |
| **The best time to start learning technical clinical skills in an undergraduate medical degree is (please tick one)**  □ Year 1 □ Year 2 □ Year 3 | | | | | |
| **What do you consider to be the advantage(s), if any, of learning technical clinical skills in year one?**  **……………………………………………………………………………………………………………………………………………………………………**  **…………………………………………………………………………………………………………………………………………………………………………………………………………………………………………………………………………………………………………………………………………** | | | | | |
| **What do you consider to be the disadvantage(s), if any, of learning technical clinical skills in year one?**  **………………………………………………………………………………………………………………………………………………………………………………………………………………………………………………………………………………………………………………………………………………………………………………………………………………………………………………………………………………………………………………** | | | | | |
